# On Tic Douloureux and Other Painful Affections of the Nerves, with Suggestions for Their Treatment by Means of the Aneuralgicon

**Published:** 1851-04

**Authors:** 


*On Tic Douloureux and other Painful Affections of the Nerves, with Suggestions for their Treatment by means of the Aneuralgicon.* By Toogood Downing, M. D. London: Churchill, 1849, pp. 73.

The author of this work is well known as a contributor to medical science, in the various periodicals in England; and the substance of his present production originally appeared in the columns of the *Lancet*. Dr. Downing says, that by means of an ingenious instrument he has constructed, he is enabled, by local treatment in the form of vapor, to give relief in most cases, and in many to cure. By means of the apparatus, the vapor from belladonna, henbane, cannabis indica, and other narcotic plants—the neuralgic organs are douched with a jet of vapor strongly impregnated with the above.

True tic douloureux is a constitutional disorder, and any means that can be had recourse to, to alleviate the intolerable sufferings, is of the utmost importance; at the same time, we cannot avoid mentioning that hundreds of cases are annually treated as constitutional, where an examination of the dental organs has at once suggested the true cause of suffering. Unfortunately for the public, medical men have little or no knowledge of the diseases of the teeth—hence their overlooking the exciting causes of pain. We, however, recommend this mode of applying the remedies, and should it, in other hands, be found to equal in its effects the results detailed in the Doctor's little work, he will have done good service to his fellow creatures. R.

